# Bacterial lipopolysaccharide-related genes are involved in the invasion and recurrence of prostate cancer and are related to immune escape based on bioinformatics analysis

**DOI:** 10.3389/fonc.2023.1141191

**Published:** 2023-04-28

**Authors:** Bangwei Che, Wenjun Zhang, Wei Li, Kaifa Tang, Jingju Yin, Miao Liu, Shenghan Xu, Tao Huang, Ying Yu, Kunyuan Huang, Zheng Peng, Cheng Zha

**Affiliations:** ^1^ School of Clinical Medicine, Guizhou Medical University, Guiyang, China; ^2^ Department of Urology, The First Affiliated Hospital of Guizhou University of Traditional Chinese Medicine, Guiyang, China; ^3^ Department of Oral and Maxillofacial Surgery, The First Affiliated Hospital of Fujian Medical University, Fuzhou, China

**Keywords:** prostate cancer, microbiome, risk score, ADT, immune infiltration

## Abstract

**Background:**

The composition of the tumor microbial microenvironment participates in the whole process of tumor disease. However, due to the limitations of the current technical level, the depth and breadth of the impact of microorganisms on tumors have not been fully recognized, especially in prostate cancer (PCa). Therefore, the purpose of this study is to explore the role and mechanism of the prostate microbiome in PCa based on bacterial lipopolysaccharide (LPS)-related genes by means of bioinformatics.

**Methods:**

The Comparative Toxicogenomics Database (CTD) was used to find bacterial LPS- related genes. PCa expression profile data and clinical data were acquired from TCGA, GTEx, and GEO. The differentially expressed LPS-related hub genes (LRHG) were obtained by Venn diagram, and gene set enrichment analysis (GSEA) was used to investigate the putative molecular mechanism of LRHG. The immune infiltration score of malignancies was investigated using single-sample gene set enrichment analysis (ssGSEA). Using univariate and multivariate Cox regression analysis, a prognostic risk score model and nomogram were developed.

**Results:**

6 LRHG were screened. LRHG were involved in functional phenotypes such as tumor invasion, fat metabolism, sex hormone response, DNA repair, apoptosis, and immunoregulation. And it can regulate the immune microenvironment in the tumor by influencing the antigen presentation of immune cells in the tumor. And a prognostic risk score and the nomogram, which were based on LRHG, showed that the low-risk score has a protective effect on patients.

**Conclusion:**

Microorganisms in the PCa microenvironment may use complex mechanism and networks to regulate the occurrence and development of PCa. Bacterial lipopolysaccharide-related genes can help build a reliable prognostic model and predict progression-free survival in patients with prostate cancer.

## Introduction

One of the most frequent malignant tumors in males is still prostate cancer (PCa) (1). According to statistics, there will be about 1.4 million new cases and 375,000 deaths worldwide in 2020 (2). Although it is believed that the occurrence and development of PCa are affected by race, family history, age, genetic susceptibility, and microenvironment, its pathogenesis is still unclear (3, 4). Although mortality has recently declined in some European countries, the long-term survival rate for metastatic PCa is less than 30% (5). The median overall survival (OS) time can currently only be slightly increased by treating metastatic anti-castration against PCa (6, 7). It is well known that PCa exhibits genomic heterogeneity. Since most prostate cancers are multifocal and different driving mutations can be active in different tumor foci, different tumor lesions in the same patient are genetically different and rarely share any somatic gene mutations, including common cancer driving genes (8). This high degree of genomic heterogeneity makes it difficult to theoretically explain the effects of different clinical statuses on tumor progression and treatment success (9).

As people pay more and more attention to the relationship between microbiota and tumors in the human microenvironment, in-depth analysis of the mutual regulation mechanism between microbiota and tumor has important clinical value and significance for the prevention and treatment of tumors (10). Recently, a large-scale study has found microbial characteristics in several types of tumors, such as gastric cancer, and lung adenocarcinoma, and shown that these microbial characteristics are unique to each type of tumor (11). In addition, earlier small-scale studies have reported different microbiome characteristics in breast, oral, prostate, and ovarian cancers (12–15). In particular, a previous study found that there were differences in microbial characteristics among different patients due to different grades and stages of PCa (16). This appears to be the same as the previously reported heterogeneity. These studies are sufficient to support the hypothesis that tumor tissues, including PCa, are likely to contain their unique microbial characteristics, and these microorganisms are likely to be involved in the occurrence and development of tumors.

However, the molecular mechanism of the prostate microbiome in PCa is still unclear. It is reported that although there are slight differences between individuals, 70% of the bacteria significantly detected in PCa samples are gram-negative (16). Lipopolysaccharide (LPS), which is also the main component of gram-negative bacteria’s cell wall, is the primary pathogenic component. LPS can elicit a strong immune response, change the morphology, metabolism, and gene expression of nearly all eukaryotic cells, promote uncontrolled expression of host cytokines, and result in severe infection (17, 18). In many studies, LPS has been shown to induce the occurrence and progression of disease by inducing the regulation of the host gene expression profile (19, 20). Here, these genes that are directly or indirectly stimulated by LPS and change their expression level are called lipopolysaccharide-related genes.

According to studies on tumors, LPS has been shown to activate TLR4 in cancer cells, which then activates NF-κB, JNK, and MAPK signals, enhancing the cancer cells’ propensity for invasion and migration (16, 21). Furthermore, LPS and LPS-induced inflammatory cytokines can increase the expression of adhesion molecules on cancer cells and endothelial cells, which in turn promotes the spread of cancer cells outside of their normal tissue. What’s more intriguing is that not all LPS are the same, according to some studies (22). For instance, LPS from E. coli typically has more inflammatory effects compared to LPS from Bacteroides species, which may help us better understand the heterogeneity of PCa. Bacteroides species produce an antagonistic form of LPS that silences pro-inflammatory signals (23, 24). Therefore, the molecular mechanism of PCa microflora is expected to be a breakthrough in the existing dilemmas of prevention and treatment.

This study examines potential molecular mechanisms of the prostate microbiome in PCa disease using genes associated with LPS. Additionally, a new prognostic model was created using the chosen LPS-related hub gene (LRHG).

## Methods

### Acquisition and preprocessing of public data

The The Cancer Genome Atlas (TCGA, https://portal.gdc.cancer.gov/) and Gene Expression Omnibus (GEO, https://www.ncbi.nlm.nih.gov) databases were used to obtain the transcriptome analysis data and corresponding clinical data for PCa, paracancerous, and normal tissues, respectively. Genotype Tissue Expression Project (GTEx) database (https://www.gtexportal.org) data were used to match normal prostate tissue transcriptome data with TCGA data. The data were standardized and subjected to a de-batch effect before matching. The GEO dataset includes GSE68555 (64 tumors, 63 adjacent normals, and 22 normals) and GSE21032 (140 tumors). GSE68555 was used to screen LRHG and perform functional analysis. A prognostic risk score model and nomogram were developed using TCGA and GTEx data, and GSE21032 was used to validate the prognostic risk model. Each sample’s Entrez gene ID needed to be converted, using the annotation platform, into the corresponding gene symbol. The average value was applied when more than one probe targeted the same Entrez gene ID. The Comparative Toxicogenomics Database (CTD, http://ctdbase.org/) was utilized to find genes associated with LPS.

### Principal component analysis and LRHG acquisition

The R software was used to analyze the transcriptome data of GSE68555 (64 tumors, 63 adjacent normal, and 22 normals) using principal component analysis (PCA). After the expression was normalized by the z-score, the reduced dimension matrix was obtained by using the prcomp function to reduce dimensionality. Finally, visualization was accomplished using the ggplot2 package.

The differentially expressed genes (DEGs) between tumor and normal (TvsN), adjacent normal and normal (TvsN), and tumor and adjacent normal (TvsAN) were found using the Limma R package, in turn. Statistical significance was defined as | logFC | > 1 and adjusted *P* < 0.05 (25). The Sangerbox online tool was then used to visualize the Venn diagram (26). The LPS-related differentially expressed genes (LDEGs) were divided into three groups. The distinct LDEGs of TvsN and ANvsN, which each overlap TvsAN, were used as the LRHG.

### Single gene enrichment analysis

GSEA software (v4.2.3) was used to conduct a gene enrichment analysis in order to discover more about the possible molecular basis of LRHG. Enrichment analysis used the expression level of LRHG as a phenotypic tag and Pearson correlation as the sorting algorithm (27).

### GeneMANIA

Using the GeneMANIA website (http://genemania.org), functionally similar genes in the LRHG were predicted, and PPI networks were constructed within them. Furthermore, it could forecast how central genes and functionally related genes will interact.

### Analysis of weighted gene co-expression network

The WGCNA R package was used in WGCNA to explore the relationship between clinical features and expression modules (28). First, the correlation between all paired genes was examined using Pearson to create the adjacency matrix. The soft threshold parameter was then set to 5 to make the co-expression network satisfy the scale-free distribution, and the dynamic tree-cutting algorithm (module size = 30) was then used to group the genes with comparable expression patterns into a single module.

### Analyzing immune cell infiltration and determining how it relates to LRHG

The assessment of immune cell infiltration was calculated based on the level of expression of immune cell-specific marker genes in the data set. 28 different types of immune cells’ marker genes were gathered from previously published articles (29). Single-sample gene set enrichment analysis (ssGSEA) was used to examine these adaptive immune cell-specific marker genes. The relationship between LRHG and the landscape of immune cell infiltration was examined using the Pearson algorithm. Using the ggplot2 R program, violin diagrams and matrix correlation heat maps are shown.

### Development and verification of prognostic risk score model

In order to create prognostic risk score models, TCGA cohort samples were categorized (30). To determine the relationship between LRHG and patients’ progression-free survival (PFS), build a Cox proportional hazard regression model, and calculate the relative contribution of LRHG to patients’ PFS. Risk score = [expression level of gene 1 × coefficient] + [expression level of gene 2 × coefficient] +… + [expression level of gene n × coefficient] was the formula we developed to predict the characteristics of genes. According to the median risk score, all samples were split into two groups: the low-risk score group and the high-risk score group. Using the survival receiver operating characteristic (ROC) R package, the time-dependent ROC curve was created in order to assess the risk score model’s correctness and examine the performance of survival prediction (31, 32). The GSE21032 dataset is used to confirm the model’s capacity for prediction.

### Clinical and immune cell infiltration and prognostic risk score correlation

The validity of prognostic risk scores based on clinical and immune characteristics was assessed using a univariate Cox regression analysis. The differences in prognostic risk scores among clinical and immune characteristics, such as age, N-stage, T-stage, gleason score, and biochemical recurrence, were assessed using a t-test or one-way ANOVA. The correlation between the prognostic risk score and immune cells was analyzed by the Pearson algorithm. *P* < 0.05 indicates that it was statistically significant.

### Drug sensitivity

In light of the significant role that androgen deprivation therapy (ADT) plays in the treatment of PCa, we used the pRRophetic R package to estimate the IC50 of bicalutamide and docetaxel and to investigate the relationship between prognostic risk score and ADT response (33, 34).

### Construction of the nomogram

*P* < 0.05 was used as the screening cutoff to examine whether the prognostic risk score and associated clinical parameters could be employed as predictors of PFS in patients with PCa using Cox regression analysis. Both the calibration curve and the decision curve analysis (DCA) were drawn to forecast performance. The rms R package was used to generate a nomogram, calibration curve, and DCA.

### Statistical analysis

R 4.1.3 was used for the statistical analysis in this study. Statistics were deemed significant at *P* < 0.05.

## Results

### Identification of LRHG connected to LPS that are differently expressed

**Figure 1:**
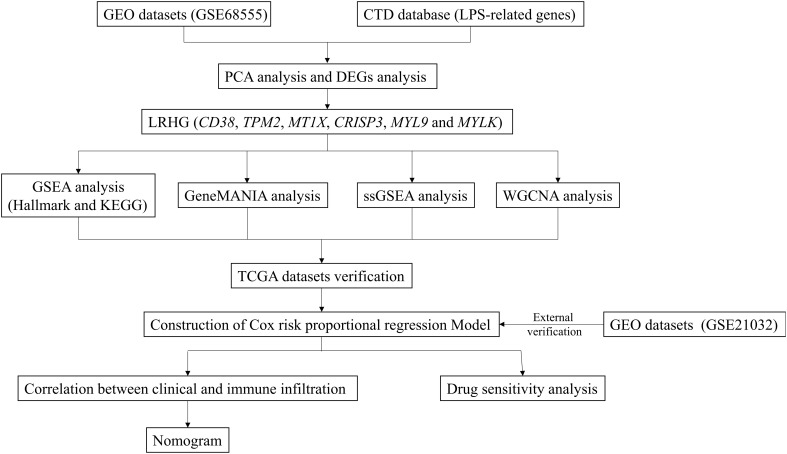
The flowchart of data collection and analyses.

**Figure 2:**
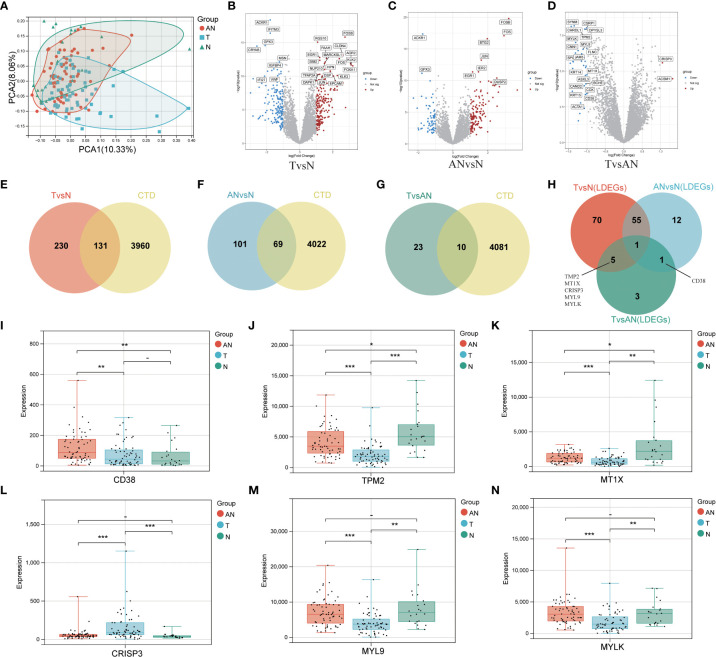
To identify differentially expressed LRHG based on GSE68555 dataset. **(A)** Principal component analysis of GSE68555; **(B)** DEGs volcano map between tumor and normal; **(C)** DEGs volcano map between the adjacent normal and normal; **(D)** DEGs volcanic map between tumor and adjacent normal; **(E–G)**. Venn diagram, TvsN DEGs in red, ANvsN DEGs in blue, TvsAN DEGs in green, and LPS-related genes obtained from CTD in yellow; **(H)** TvsN, ANvsN and TvsAN LDEGs Venn diagrams; **(I–N)**. The expression of *CD38*, *TPM2*, *MT1X*, *CRISP3*, *MYL9* and *MYLK* in prostate cancer, adjacent normal and normal, * *P* < 0.05; * * *P* < 0.01; *** *P* < 0.001. -: no relevant data.

The general workflow of this study is displayed in **Figure 1**. We performed PCa analysis on the GSE68555 dataset. The findings revealed that the compositions of the adjacent normal (AN), which was in between the tumor’s (T) and normal’s (N) compositions, were both comparable and distinct (**Figure 2A**). The gene differences between T and N, AN and N, and T and AN were analyzed using adjusted *P<*0.05 and | logFC | > 1, and then overlapped with lipopolysaccharide-related genes, yielding 131 (TvsN), 69 (ANvsN), and 10 (TvsAN) LDEGs, respectively (**Figures 2B–G**). Then the LDEGs of TvsN and ANvsN were overlapped with TvsAN LDEGs, respectively, and 6 differentially expressed LRHG genes were obtained, namely *CD38*, *TPM2*, *MT1X*, *CRISP3*, *MYL9*, and *MYLK* (**Figure 2H**). Additional research on LRHG expression in T, AN, and N. When compared to T and N, the expression level of *CD38* in AN was considerably greater (*P* < 0.05). *TPM2*, *MT1X*, *MYL9*, and *MYLK* expression levels were considerably lower in T than in AN and N, while CRISP3 was on the contrary (*P <*0.05). (**Figures 2I–N**). On the basis of this, we proposed that these LRHG may control tumor invasion, with *CD38* playing a key role in AN.

### Analysis of enrichment for differentially expressed LRHG

**Figure 3:**
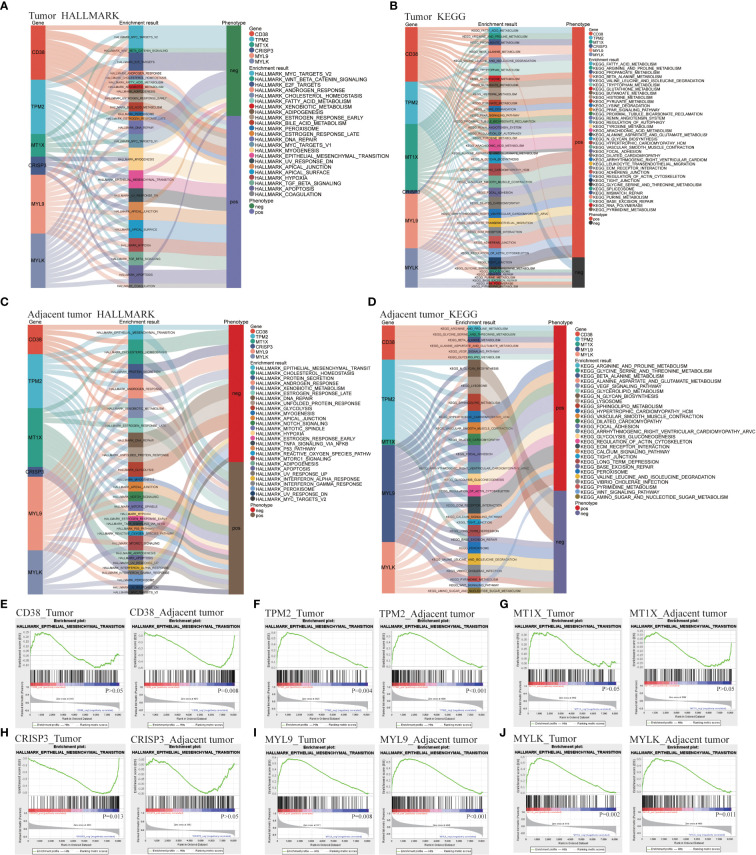
LDEGs functional analyses. **(A, B)** HALLMARK and KEGG enrichment analysis of LRHG in tumor. **(C, D)**. HALLMARK and KEGG enrichment analysis of LRHG in adjacent normal; **(E–J)**. The enrichment results of LRHG in EMT function.

Hallmark and Kyoto encyclopedia of genes and genomes (KEGG) function enrichment were carried out in T and AN, respectively, to investigate the associated LRHG functions. The functional phenotypes associated with LRHG in T were primarily focused in tumor invasion, lipid metabolism, sex hormone response, DNA repair, and apoptosis, according to Hallmark enrichment analysis. Particularly, MYC TARGETS V1/V2, WNT BETA CATENIN SIGNALING, E2F TARGETS, and ANDROGEN RESPONSE were involved in the transition from androgen reliance to androgen non-dependency (**Figure 3A**). The functional phenotypes associated with LRHG were concentrated in a number of metabolic pathways, including FATTY ACID METABOLISM and ARACHIDONIC ACID METABOLISM, according to KEGG enrichment analyses (**Figure 3B**). The functional phenotypes associated with LRHG in AN were primarily focused in cancer, invasion, sex hormone response, and DNA repair, according to HALLMARK enrichment analysis (**Figure 3C**). The functional phenotypes linked to LRHG were concentrated on numerous amino acid metabolic pathways and transfer-related pathways, according to KEGG enrichment analysis (**Figure 3D**). In addition, all five of the other LRHG, with the exception of *MT1X*, were linked to the epithelial mesenchymal transition (EMT) phenotype. *TPM2*, *MYL9*, and *MYLK* are substantially linked with the EMT phenotype in both T and AN, while *CD38* and *CRISP3* were the functional phenotypes most concentrated in AN or T, respectively (**Figures 3E–J**).

### Prediction of coexpression network of LRHG on GeneMANIA

GeneMANIA was used to predict the co-expression network and function of LRHG, and 20 additional co-expression genes were obtained (**Supplement Figure 1**). Their functions were concentrated on actin cytoskeleton, myosin complex, muscle contraction, contractile fiber and actomyosin. These functions were closely related to the above tumor invasion and EMT phenotype. The function of the LRHG in tumor invasion and metastasis was verified.

### LRHG and tumor aggressive phenotype

**Figure 4:**
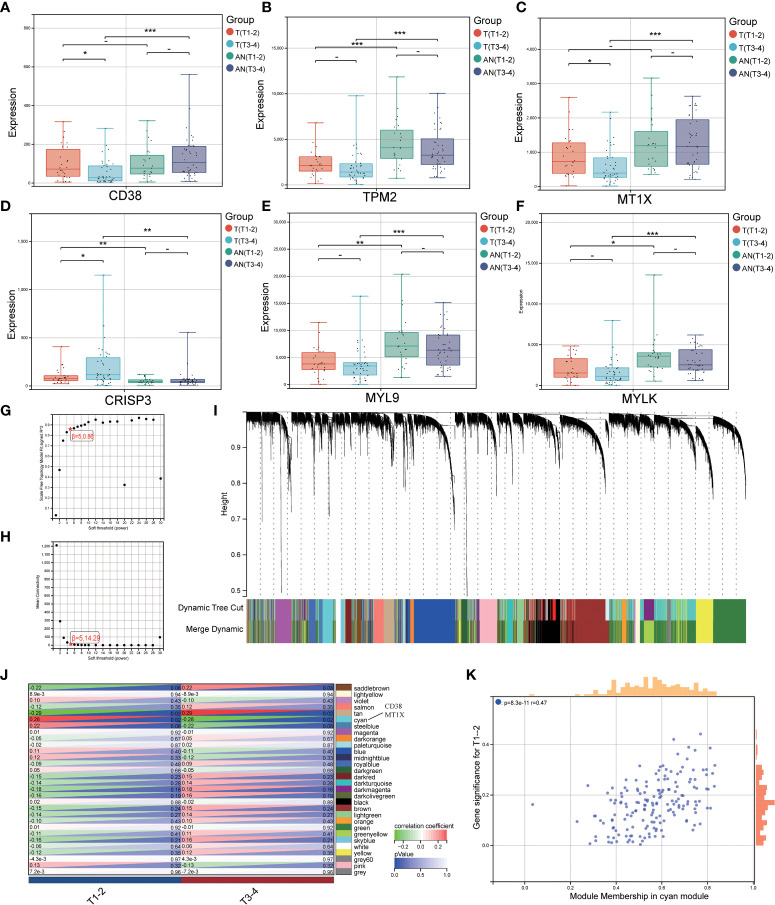
LRHG and tumor invasion phenotype. **(A–F)**. Expression of *CD38*, *TPM2*, *MT1X*, *CRISP3*, *MYL9*, and *MYLK* in non-invasive (T1-2) and invasive (T3-4) tissues; **(G, H)**. Scale-free fitting Index and average Connectivity Analysis of different soft threshold Power (β); **(I)**. The clustering tree diagram of the recognition module. Each module is given a separate color as a logo, including 30 different modules; **(J)** The heat map of module-characteristic relationship and cyan module were significantly correlated with prostate cancer invasion; **(K)** The scatter diagram of the correlation between the number of gene module members and gene significance in the cyan module. * *P* < 0.05; * * *P* < 0.01; *** *P* < 0.001. -: no relevant data.

We investigated the expression of LRHG in invasive PCa (T3-4) and non-invasive PCa (T1-2) to further investigate the connection between LRHG and tumor invasion. Only *CD38*, *MT1X*, and *CRISP3* showed differences in expression levels between non-invasive and invasive tumors in T. And *CD38* and *MT1X* expression levels in invasive tumors were significantly lower than in non-invasive tumors, whereas *CRISP3* was the opposite (**Figures 4A–F**). Then, WGCNA was constructed from T expression data to verify the relationship between LRHG and tumor invasion, and the soft threshold β = 5 (scale-free R ^ 2 = 0.86) was selected (**Figures 4G, H**). A total of 30 modules were screened, and the cyan module showed a statistically significant correlation with non-invasive PCa (*P* < 0.05) (**Figures 4I–K**). *CD38* and *MT1X* genes were found in the cyan module.

### Evaluation of LRHG and immune microenvironment

**Figure 5:**
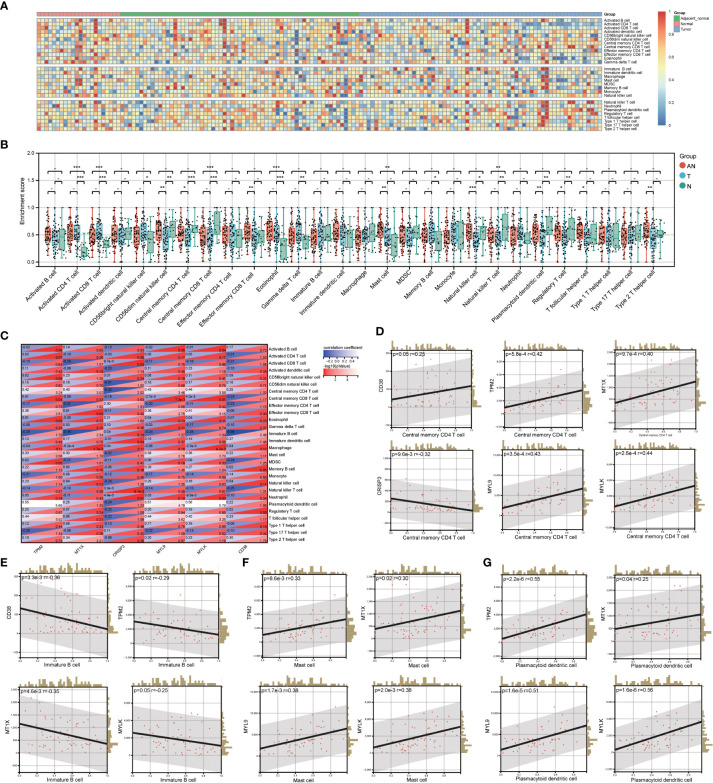
Evaluation of LRHG and immune microenvironment. **(A)** Heatmap of the landscape of 28 immune cell subpopulations infiltration in prostate; **(B)** Comparison of immune cell composition in Tumor, adjacent normal and nomal; **(C)** Heat map of the relationship between LRHG and 28 immune cells; **(D-G)**. The correlations between LRHG and Central memory CD4 T cell, Immature B cell, Mast cell, Plasmacytoid dendritic cell, respectively. * *P* < 0.05; * * *P* < 0.01; *** *P* < 0.001. -: no relevant data.

By investigating the GSE68555 data set, the infiltration landscape of 28 different immune cell types in PCa was examined (**Figure 5A**). Activated CD4^+^ T cells, activated CD8^+^ T cells, central memory CD8^+^ T cells, eosinophils, and natural killer T cells did not differ substantially between T and AN (*P* > 0.05) according to the analysis of variance approach, but they did differ significantly from N (*P* < 0.05). CD56dim natural killer cells, central memory CD4^+^ T cells, natural killer cells, plasmacytoid dendritic cells, and regulatory T cells did not substantially differ between AN and N (*P* > 0.05), although AN and N did significantly differ from T (*P*<0.05). Mast cell differences between T and N were not significant (*P* > 0.05), whereas those between T and N and AN were (*P*<0.05). (**Figure 5B**). The relationship between LRHG and the degree of immune cell subset infiltration was next examined (**Figure 5C**). Central memory CD4^+^ T cell were significantly correlated with LRHG (6/6), including a positive correlation with *CRISP3* and a negative correlation with others (*P* < 0.05) (**Figure 5D**). Immature B cell, mast cell, and plasmacytoid dendritic cell were significantly associated with LRHG (4/6) (*P* < 0.05) (**Figures 5E–G**).

### Develop prognostic risk characteristics

**Figure 6:**
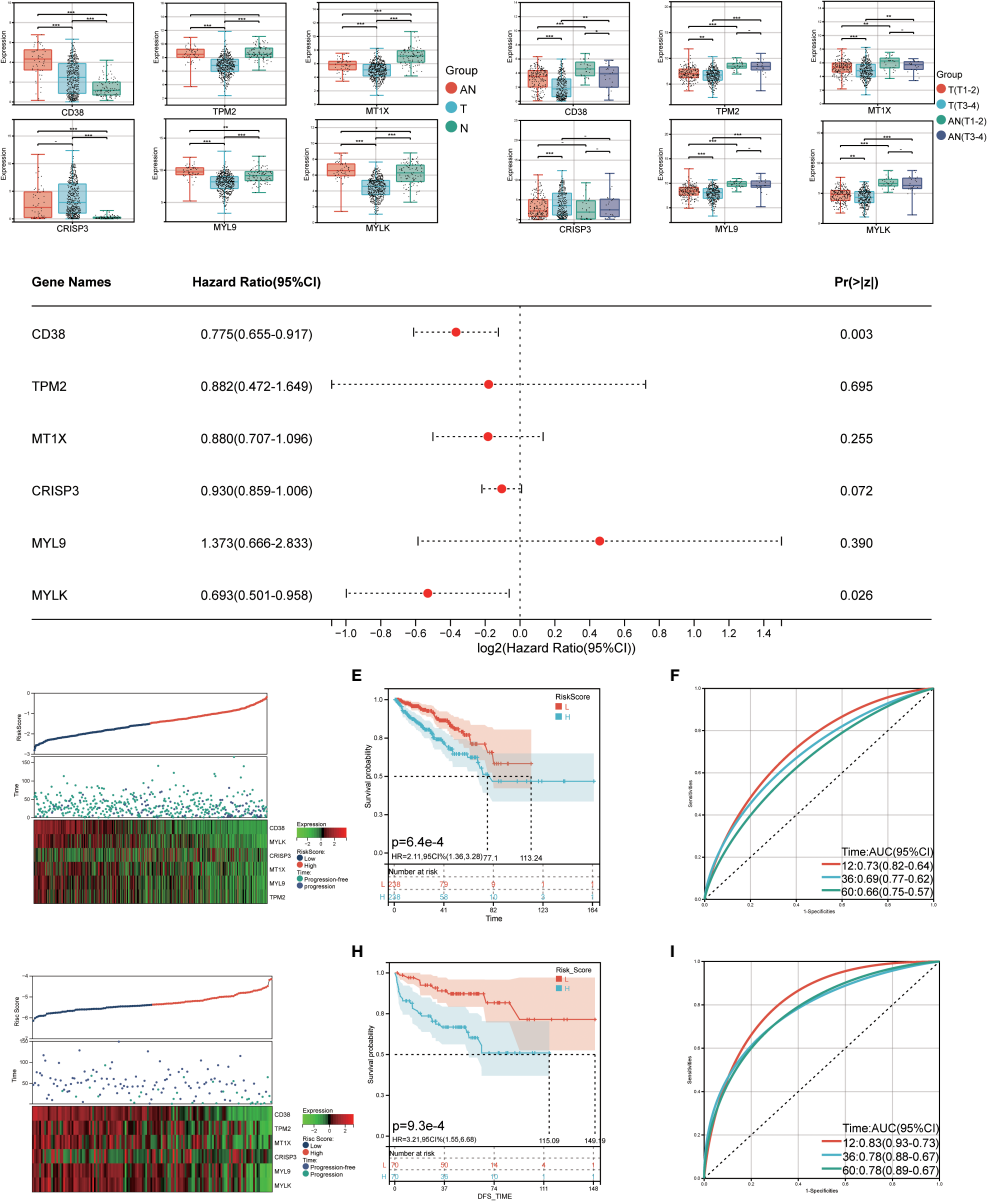
Construction of prognostic risk model. **(A)** expression level of LRHG in TCGA cohort in T, AN and N; **(B)** Expression of LRHG in TCGA cohort in non-invasive prostate cancer and invasive prostate cancer; **(C)** Forest map of multivariate Cox model of LRHG in risk score; **(D)** Expression of LRHG in TCGA cohort in low risk and high-risk population; **(E)** TCGA low-risk and high-risk population progression-free survival curve; **(F)** Time-dependent ROC curve of TCGA cohort; **(G)** Expression of LRHG in GSE21032 cohort in low risk and high risk population; **(H)** Progression-free survival curve of low-risk and high-risk population in GSE21032 cohort; **(I)** Time-dependent ROC curve of GSE21032 cohort. * *P* < 0.05; * * *P* < 0.01; *** *P* < 0.001. -: no relevant data.

The TCGA cohort was used to verify the LRHG expression level prior to model construction. The findings demonstrated that the TCGA cohort’s distribution trend for LRHG expression in T, AN, and N was consistent with that of the GSE68555 cohort (**Figures 6A, B**). Then, for 476 PCa patients with data on PFS, a Cox risk proportional regression model was created (**Figure 6C**): The risk score was calculated as follows: [expression level of *CD38* × 0.774989] + [expression level of *TPM2* × 0.882436] + [expression level of *MT1X* × 0.880267] + [expression level of *CRISP3* × 0.929862] + [expression level of *MYL9* × 1.373418] + [expression level of *MYLK* × 0.692560]. According to the median risk score, patients with PCa were equally divided into two groups: the low-risk score group and the high-risk score group. The number of PCa progressions in the high-risk group was significantly higher than that in the low-risk score group, and the heat map showed differential expression of LRHG between the two groups (**Figure 6D**). The overall PFS in the low-risk group was significantly longer than that in the high-risk group, according to the survival curve (*P*<0.05) (**Figure 6E**). The model’s dependability was further demonstrated by the time-dependent ROC curve, which had AUC values for the 1-, 3-, and 5-year periods of 0.73, 0.69, and 0.66, respectively (**Figure 6F**). The prognostic risk model’s universality was examined using the GSE21032 cohort. The overall PFS of the low-risk group was much longer than that of the high-risk group, which is consistent with the TCGA cohort (*P*<0.05). (**Figures 6G, H**). The time-dependent ROC’s 1-, 3-, and 5-year AUC values were 0.83, 0.78, and 0.78, respectively (**Figure 6I**).

### Clinical/immune cell infiltration and risk score

**Figure 7:**
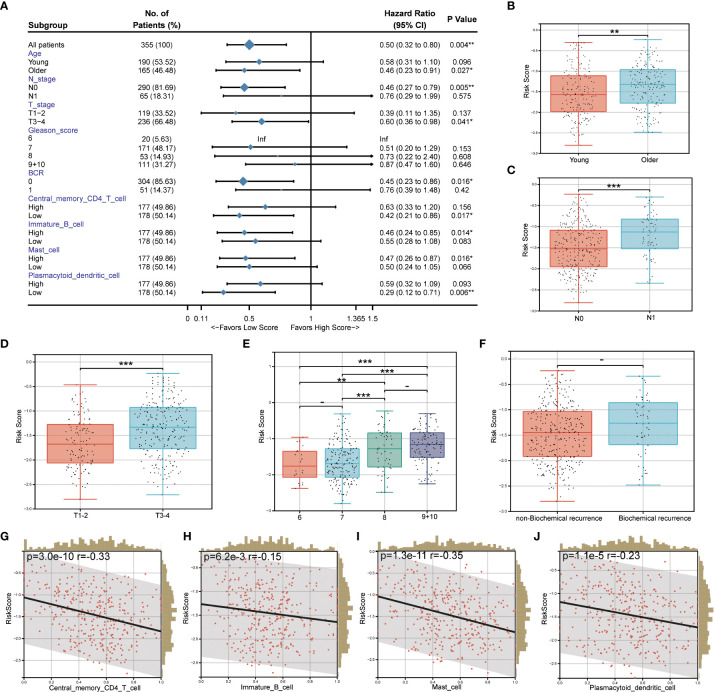
Correlation between prognostic risk score and clinical/immune characteristics. **(A)** Univariate Cox regression analysis of prognostic risk score and clinical feature subgroup/four kinds of immune cells; **(B–F)**. Differences in prognostic risk scores among clinical feature subgroups; **(G–J)**. Correlation between four kinds of immune cells and prognostic risk score. * *P* < 0.05; * * *P* < 0.01; *** *P* < 0.001. -: no relevant data.

The relationship between the prognostic risk score and clinical and immune characteristics was then further evaluated using the TCGA cohort. 355 patients were included after the samples were cleaned of any missing clinical characteristic data. According to the findings, patients with advanced age, invasive PCa without lymph node metastasis, no biochemical recurrence, low central memory CD4^+^ T cell infiltration, high immature B cell infiltration, high mast cell invasion, and low plasmacytoid dendritic cell invasion all significantly benefited from having a low-risk score (**Figure 7A**). It was discovered that, with the exception of biochemical recurrence, there were significant differences in the prognostic risk scores among the various clinical feature subgroups (*P*<0.05) (**Figures 7B–F**). Additionally, there was a substantial negative correlation between the four different kinds of immune cells and the prognostic risk score (*P*<0.05) (**Figures 7G–J**).

### Drug sensitivity

We also examined the relationship between prognostic risk scores and popular therapeutic medications (bicalutamide and docetaxel) because GSEA enrichment analysis suggested that these LRHG may contribute to the development of androgen-dependent PCa into an androgen-independent condition. The findings revealed that docetaxel had a lower IC50 in the high-risk group and bicalutamide had a lower IC50 in the low-risk group (**Supplementary Figures 2A, B**). The *AR* mRNA showed similar results, with PCa being significantly more expressed in the low-risk group than the high-risk group (**Supplementary Figure 2C**). As a result, it seems possible that the prognostic risk score could be helpful in determining how to use medications.

### Establishment of nomogram

**Figure 8:**
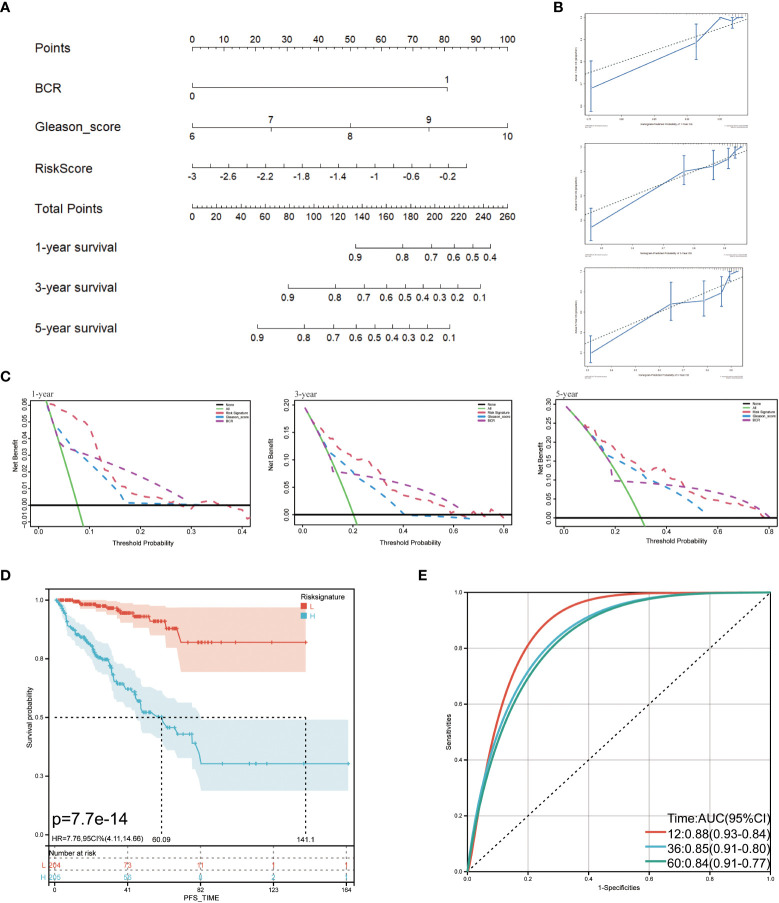
Predictive value of prognostic risk score combined with clinical/immune characteristics for PFS in patients with TCGA cohort. **(A)** Nomogram for predicting PFS of patients in TCGA cohort; **(B)** The calibration diagram of the nomogram. The X axis is the survival rate predicted by the nomogram, and the Y axis is the actual survival rate; **(C)** Analysis of decision curve of nomogram; **(D)** Survival curve of low-signature and high-signature; **(E)** Time-dependent ROC curve.

The TCGA cohort’s clinical and immune characteristics, as well as the relationship between PFS and the prognostic risk score, had to be assessed (**Table 1**). The PFS was finally predicted using a nomogram that combined biochemical recurrence, Gleason score, and risk score (**Figure 8A**). The nomogram can successfully predict PFS in PCa patients, according to 1-, 3-, and 5-year correction curves (**Figure 8B**). The DCA over 1-, 3-, and 5-year reveals that the model offers greater benefits than the Gleason score. When the values of 1-, 3-, and 5-year were 0-0.13, 0-0.3, and 0-0.5, respectively, the benefit of threshold probability was significantly greater than that of biochemical recurrence (**Figure 8C**). Additionally, the K-M Survival Curve demonstrated that low-signature individuals had significantly longer PFS times than high-signature individuals (**Figure 8D**). The 1-, 3-, and 5-year AUC values for the time-dependent ROC were 0.88, 0.85, and 0.84, respectively, showing that it was more predictive than the single prognostic risk score model (**Figure 8E**).

**Table 1:** Univariate and multivariate Cox regression analysis of risk score and other clinicopathological factors for PFS in TCGA cohort.

character	Univariate analysis			Multivariate analysis		
	HR	95% CI	P value	HR	95% CI	P value
Age	1.03	1.00-1.07	0.039^*^	1.04	1.00-1.08	0.058
N stage	1.85	1.13-3.03	0.014^*^	0.98	0.58-1.65	0.925
T stage	3.61	2.03-6.42	<0.001^***^	1.3	0.63-2.67	0.481
Gleason score	2.21	1.77-2.75	<0.001^***^	1.58	1.18-2.12	0.002^**^
Biochemical recurrence	8.00	5.18-12.36	<0.001^***^	4.37	2.68-7.12	<0.001^***^
Risk Score	2.72	1.82-4.06	<0.001^***^	1.68	1.06-2.67	0.026^*^
Central memory CD4 T cell	1.07	0.43-2.65	0.884	–	–	–
Immature B cell	1.7	0.64-4.52	0.288	–	–	–
Mast cell	0.77	0.32-1.90	0.576	–	–	–
Plasmacytoid dendritic cell	2.51	1.00-6.3	0.051	–	–	–

* P < 0.05; ** P < 0.01; *** P < 0.001. -: no relevant data.

## Discussion

The variety of microorganisms in tumors and the depth of their effects have not yet been fully understood due to technological limitations, but they are involved in every stage of a tumor’s development, including its occurrence, development, metastasis, and immune response, particularly PCa. Consequently, it might be one of the best ways to use bioinformatics to investigate and forecast the role of the prostate microbiome in PCa. In this study, we used the LPS-related genes obtained by CTD as the basis for our analysis and used the GEO data set to identify LRHG differential genes related to LPS. We can then investigate the molecular mechanisms of the prostate microbiome involved in PCa by combining bioinformatics analysis such as functional enrichment of LRHG, immune infiltration correlation, and co-expression network construction, and the prognostic risk model and nomogram were created. Their significance in PCa progression was then confirmed in the verification and test cohorts obtained from TCGA, GTEx, and GEO.

For this study, the LRHG genes *CD38*, *TPM2*, *MT1X*, *CRISP3*, *MYL9*, and *MYLK* were obtained. *CD38* participates in the typical activities of cell surface receptors, such as signal transduction of activation and proliferation events and atypical cell adhesion. In the event of bacterial LPS stimulation, it plays a defensive role and regulates the immune system against bacterial infection. The biological functions of *TPM2*, *MT1X*, and *CRISP3* stimulated by LPS are not clear. The current evidence is that an increase in their expression level was observed in the transcriptional group of peripheral blood immune cells. Both *MYL9* and *MYLK* encode myosin light chain protein, which can increase the permeability of epithelial cells and promote the progression of inflammation in the inflammation produced by bacterial LPS.

In previous studies on tumors, LRHG has been shown to be closely related to tumors. Recent research, in particular, suggests that *CD38* may represent a novel immunosuppressive target for PCa. Immune progenitor cells express *CD38*, a ribosyl cyclase family extracellular enzyme, on their surface. Its receptors, ligands, and enzymes contribute to the growth and dissemination of malignancies by regulating immune response response, metabolism, calcium-mediated signal transduction, cell adhesion, and migration (35, 36). The enzyme activity of *CD38* is to catalyze the conversion of NAD^+^ to ADPR, cADPR, NAM, and other metabolites. This activity is very important for maintaining the dynamic balance of NAD, nicotinamide, and other substances in the body (37). It is also worth noting that, unlike most tumors, *CD38* has been detected to be down-regulated in PCa, especially in advanced castration-resistant prostate cancer (CRPC), which may be due to methylation silencing (38, 39). Additionally, some studies have revealed that invasive PCa and unfavorable results are frequently linked to decreased *CD38* expression (40). Although the other five LRHG in PCa have not received the same amount of research as CD38, it is known that they may also contribute to the development and spread of PCa. One of *Tm1*’s variants, *TPM2*, is involved in the synthesis of cytoskeletal tropomyosin. *Tm1*, a tumor suppressor, is said to be able to stop the growth of tumors and change the phenotype of transformed cells (41). *MT1X* is a member of the metallothionein (MT) family, which controls metal ion homeostasis to influence tumor growth, progression, metastasis, and drug resistanc*e* (42). *CRISP3* belongs to a large family of cysteine-rich secretory proteins and is essential for the development of invasive PCa *in vivo* from carcinoma *in situ* as well as for AR-independent transcriptional processes (43, 44). Both *MYL9* and *MYLK* belong to myosin light chain proteins, which can promote the growth and metastasis of PCa and participate in the immune infiltration of PCa (45, 46). This is confirmed by the results of the GSEA analysis. These LRHG are significantly associated with invasion, fat metabolism, sex hormone response, DNA repair, and apoptosis in tumors. It may also play a role in the transformation of androgen-dependent PCa into androgen-independent PCa. At the same time, we also explored the co-expression network where LRHG is located, and its functions are mainly focused on actin cytoskeleton, myosin complex, muscle contraction, contractile fiber, and actomyosin, which are closely related to tumor invasion and the EMT phenotype. In particular, *CD38* and *MT1X* are more likely to be the key genes in this co-expression network.

Immune cell research and clinical use have given hope for a number of cancers, but PCa patients have not yet benefited from it. One of the key causes is that PCa has a clear immunosuppressive microenvironment (47). Consequently, we also looked into the connection between LRHG and the pattern of immune infiltration in PCa. Our results show a significant relationship between LRHG and the degree of immune cell infiltration in the tumor. Tumors can evade the immune system by promoting the depletion of mast cells, immature B cells, central memory CD4^+^T cells, and plasma cell-like dendritic cells. As upstream and intermediary cells in the immune response, central memory CD4^+^T cells are a subtype of central memory T cells (Tcm) (48). Tcm is in charge of long-term memorization of the tumor antigen in the tumor microenvironment following immune system recognition of the tumor antigen (49). A large number of effector memory T cells (Tem) that target the tumor continue to be produced in response to the stimulation of the tumor antigen and then differentiate into a large number of effector T cells that kill cancer cells (50). By presenting tumor antigens, mast cells and plasmacytoid dendritic cells can encourage the differentiation of B and T lymphocytes (51–53).

At present, antibody-based immunotherapeutic drugs mainly target immune checkpoints, such as CTLA-4, PD-1, and LAG-3 (54, 55). Cancer cells turn off the immune system’s immune response to cancer by hijacking these checkpoint proteins. However, in this study, the cause of immunosuppression in the prostate cancer microenvironment may be due to the depletion of antigen-presenting cells. As a result, drugs targeting T-cell immune checkpoints for prostate cancer often fail to achieve the desired results (56). It is reasonable to believe that cellular immunotherapy, which controls the immune osmotic mode of tumors by affecting antigen presenting cells, is more likely to benefit PCa patients than traditional immunotherapy. Cellular immunotherapy using antigen-presenting cells (sipuleucel-T) has been shown to prolong the survival time of CRPC patients (57).

In light of this, we posit that a complex web of mechanisms may be used by microorganisms in the PCa microenvironment to contribute to the emergence and development of PCa. The overall impact of this intricate network of mechanisms determines how PCa develops. Based on these LRHG, we developed a risk score model to assess the effects of gene co-expression thoroughly. Our hypothesis was then confirmed using univariate and multivariate Cox regression analysis to predict PFS of PCa. The survival curve revealed that individuals with high-risk scores had a considerably poorer outcome for PCa and shorter PFS, and shorter PFS was seen in these patients. A low-risk score has a protective effect on patients in the study of the clinical and immune characteristics of PCa, especially those who are older, have invasive PCa, no lymph node metastasis, no biochemical recurrence, low central memory CD4^+^ T cell infiltration, high immature B cell infiltration, high mast cell infiltration, and low plasmacytoid dendritic cell infiltration.

The results of the preliminary analysis of the androgen response showed that the *AR* mRNA expression was higher in the low-risk group. This implies that PCa patients may respond differently to androgen therapy depending on their risk scores. This might also be true. Low-risk PCa patients showed a lower estimated IC50 for bicalutamide, whereas high-risk PCa patients showed a lower estimated IC50 for docetaxel. This further suggests that PCa patients’ drug selection is influenced by their risk score.

Additionally, the risk score and the screened clinical and immunological parameters were combined to construct a nomogram. The nomogram has a better predictive value than a different risk score model, according to a survival analysis. The calibration curve and ROC curve also demonstrate the nomogram’s high accuracy. According to DCA, the nomogram was preferable to the Gleason score for patients. The development of the model initially demonstrates the potential for PCa patients, particularly those with non-metastatic castration-resistant prostate cancer (nmCRPC), to greatly benefit from research based on PCa microorganisms.

The limitations of this study must be acknowledged, even though these findings point in a new direction for a deeper investigation of the molecular mechanisms underlying PCa. First of all, current animal and cell experiments cannot accurately simulate the microbial microenvironment in the tumor due to technical limitations in our ability to fully detect the composition and structural characteristics of PCa microorganisms. Second, the underlying mechanism governing the PCa process is still unknown for the co-expression network of these LRHG and other related genes. More research into their biological functions must be done through carefully thought-out experiments. Last but not least, this study is primarily based on genes associated with LPS that the CTD identified after reviewing expression data from public databases. As a result, the study’s findings have some limitations because it is clear that PCa may also be caused by other pathogenic elements of the PCa microbiome. This suggests that additional molecular mechanisms require study.

There are still opportunities and challenges in the diagnosis and treatment of prostate cancer (58–60). Although the microbiome is still in its infancy in the study of prostate cancer based on various factors, this does not prevent it from becoming an attractive and promising research direction. In the future, with the in-depth study of the microbiome in prostate cancer, it may be accompanied by the use of microorganisms as a diagnostic tool for prostate cancer, microbial-based prevention, and immunotherapy (61). All these are worthy of our expectations and efforts.

## Conclusion

In conclusion, our study shows that a complex network of mechanisms involving microbes contributes significantly to the emergence and development of PCa. The overall impact of this intricate network of mechanisms may have an impact on PCa development. This could aid in our understanding of the PCa’s underlying molecular mechanisms. However, at the present technical level, it is challenging to carry out a thorough and in-depth study through experiments. Finally, this study shows that bacterial LPS-related genes can help establish reliable prognostic models and predict PFS in patients with prostate cancer. In addition, the study raises the possibility that cellular immunotherapy may improve the prognosis of patients with PCa.

## Data availability statement

Publicly available datasets were analyzed in this study. This data can be found here: TCGA (https://portal.gdc.cancer.gov/); GTEx(https://www.gtexportal.org); GSE68555(https://www.ncbi.nlm.nih.gov/geo/query/acc.cgi?acc=GSE68555); GSE21032(https://www.ncbi.nlm.nih.gov/geo/query/acc.cgi?acc=GSE21032.

## Author contributions

Study concept and design: KT. Collection and assembly of data: BC, WZ, and WL Data analysis and interpretation: BC, WZ, and JY. Manuscript revised: BC, WZ, ML, SX, and TH. Manuscript writing and review: BC, WZ, and WL. YY, KH, ZP, and CZ participated in the discussion. All authors contributed to the article and approved the submitted version.

## Supplementary material

Supplementary Figure 1 Prediction of coexpression network of LRHG on GeneMANIA.

Supplementary Figure 2 Correlation between risk score and sensitivity of bicalutamide and docetaxel based on TCGA cohort (n=476). **(A)** Bicalutamide drug sensitivity; **(B)** Docetaxel drug sensitivity; **(C)** *AR* mRNA expression. * *P* < 0.05; * * *P* < 0.01; *** *P* < 0.001.
