# Supplementary figures and images for: Bacterial lipopolysaccharide-related genes are involved in the invasion and recurrence of prostate cancer and are related to immune escape based on bioinformatics analysis
Source: Front Oncol. 2023 Apr 28;13:1141191. doi: 10.3389/fonc.2023.1141191 (PMC10175693; doi:10.3389/fonc.2023.1141191)

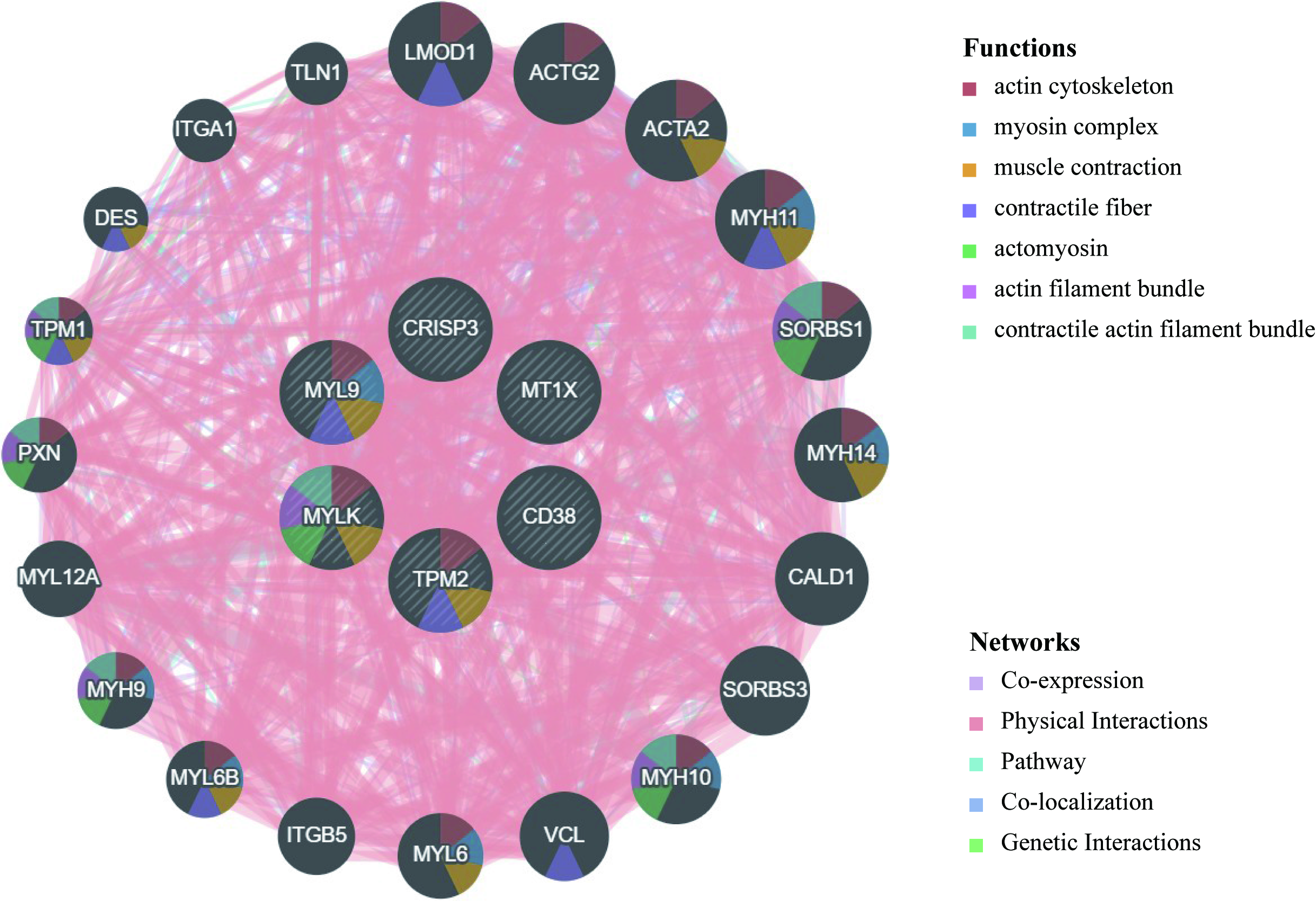

Supplement: Supplementary Figure 1 — Prediction of coexpression network of LRHG on GeneMANIA. [file Image_1.tif]

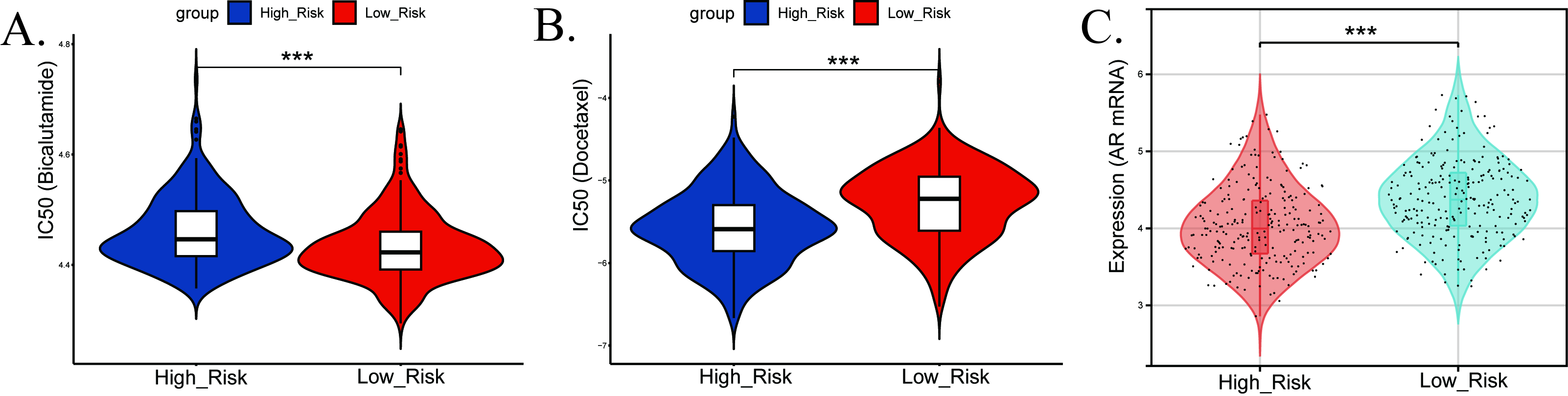

Supplement: Supplementary Figure 2 — Correlation between risk score and sensitivity of bicalutamide and docetaxel based on TCGA cohort (n=476). (A) Bicalutamide drug sensitivity; (B) Docetaxel drug sensitivity; (C) AR mRNA expression. * P < 0.05; * * P < 0.01; *** P < 0.001. [file Image_2.tif]
